# From Misdiagnosis to Discovery: Expanding the Spectrum of Spliceosomopathies

**DOI:** 10.3390/genes17091020

**Published:** 2026-08-27

**Authors:** Giulia Bruna Marchetti, Francesca Cappuccini, Lucrezia Goisis, Elvira Verduci, Federica Natacci, Cristina Gervasini, Valentina Massa, Donatella Milani, Maria Iascone, Laura Pezzoli

**Affiliations:** 1 Fondazione IRCCS Ca’ Granda Ospedale Maggiore Policlinico di Milano, 20122 Milan, Italy; 2 Laboratorio Genetica Medica, ASST Papa Giovanni XXIII, 24127 Bergamo, Italy; 3 Scuola di Specializzazione in Genetica Medica, Università degli Studi di Milano Bicocca, 20126 Milan, Italy; 4 Scuola di Specializzazione in Genetica Medica, Università degli Studi di Milano, 20122 Milan, Italy; 5 Department of Health Sciences, Università degli Studi di Milano, 20122 Milan, Italy; 6 “Aldo Ravelli” Center for Neurotechnology and Experimental Brain Therapeutics, Università degli Studi di Milano, 20122 Milan, Italy

**Keywords:** *PUS7*, misdiagnosis, neurodevelopmental disorder

## Abstract

Background: Defining a genetic diagnosis is a challenging and stepwise process, often limited by current knowledge perspectives. Alterations in non-coding RNAs and in their modulating mechanisms are now emerging as one of the main possible explanations underpinning many unsolved cases of neurodevelopmental disorders. The latest description concerns the *PUS7* gene, which encodes a pseudouridine synthase (PUS) enzyme, which has been causatively associated with an extremely rare recessive disease characterized by postnatal microcephaly, neurodevelopmental impairment, and minor dysmorphisms. Here, we report a novel case of this poorly known disorder, which was diagnosed in a young girl referred to our center following a misdiagnosis of Kleefstra Syndrome type 2. Methods: Whole-genome sequencing detected two composite heterozygous deletions in the *PUS7* gene, which led us to dismiss the former diagnosis. Guided by the gestaltic evaluation of our case, we reviewed the current knowledge about the *PUS7*-related disorder and, driven by the biological role of this enzyme, investigated its similarities with a main spliceosomopathy, ReNU syndrome. Results: Data obtained from this exploratory analysis, together with the recent evidence from the literature, support the affinity between these conditions, suggesting that disorders of pseudouridylation might possibly be included among spliceosomopathies. Conclusions: Aside from expanding the current knowledge about the *PUS7*-related disorder and exploring its nosological classification, one of the main aims of our report is to raise awareness of the impact of genetic labelling and misdiagnoses. The use of whole-genome sequencing technologies requires the effective collaboration between laboratories and physicians to reach the correct diagnosis.

## 1. Introduction

Alterations in gene expression are, to date, considered a primary cause of human diseases, although their regulatory processes are still poorly understood [1]. Similarly, genomic data analyses can still yield inconclusive results, whether negative or uncertain, marking the boundaries of our current knowledge in this field. This interpretive complexity could easily conceal pitfalls and generate wrong diagnoses, which are eventually corrected by emerging technologies and new knowledge. A compelling new perspective highlights RNA perturbations, alongside DNA changes, as a key mechanism underpinning human diseases because of their crucial role in fine-tuning gene expression [2]. The association between non-coding RNAs and neurodevelopmental disorders (NDDs) has represented one of the more important breakthroughs in the syndromic field over the last few years [3]. A prominent example is ReNU syndrome which has been estimated to account for nearly 0.4% of all NDDs [3,4]. Following the first description of ReNU syndrome, a larger family of neurodevelopmental disorders determined by alterations in noncoding RNAs’ genes involved in the spliceosome creation have been reported, expanding the number of Spliceosomopathies (SPPs). Among post-transcriptional RNA modifications, pseudouridylation is one main mechanism mediated by pseudouridine synthase (PUS) enzymes [5]. Although its precise biological significance remains incompletely understood, defects in PUS proteins (e.g., PUS1, PUS3, and PUS7) have been related to a large variety of human diseases, ranging from NDD to cancer [6,7,8]. The *PUS7* gene, in particular, has been recently associated with an ultrarare recessive form of NDD, formally defined as “Intellectual developmental disorder with abnormal behaviour, microcephaly, and short stature”, IDDABS, (MIM #618342). Since 2018, only 13 affected patients have been described in the literature [9,10,11,12,13]. Here, we report a novel case of this ultrarare condition diagnosed through an extensive whole-genome analysis, after an initial wrong diagnosis of a *KMT2C*-related disorder. Along with discussing the reasons for the initial misdiagnosis and reviewing the available information regarding IDDABS, we will attempt to define through Artificial Intelligence (AI) technologies the gestaltic profile of this poorly known condition. Furthermore, driven by biological and phenotypical overlaps, we will discuss the possible inclusion of this extremely rare condition within the larger and novel family of SPPs.

## 2. Case Report

The patient was the first child of a healthy unrelated couple, conceived through an ovodonated twin pregnancy. A caesarean section was scheduled for 36 gestational weeks due to twinning. Both girls showed normal auxological parameters (BW: 2300 g, BL: 47 cm, OFC: 32.5 cm). At birth, multiple ventricular septal defects were detected, which resolved spontaneously over years. At the age of 8 months, she underwent the surgical correction of metopic suture synostosis to address an evident trigonocephaly. She later presented with plagiocephaly, hypotonia, and a delay in developmental milestones. A Griffiths evaluation at the age of 4 years assessed a score below 24 months, while brain MRI revealed an underdeveloped left parietal bone and a thin corpus callosum. Growing up, she suffered from a refractive defect, strabismus, and hypoacusia, and presented with flat feet and kyphosis. The age of menarche was 11 years, but menstruation was irregular (polymenorrhoea). Finally, constipation was reported. According to the family, no behavioural problems arose.

At the age of 11 years, she first accessed our Centre for a genetic evaluation in the context of a research project dedicated to “Kleefstra Syndrome” (see below). A physical examination revealed weight and height at higher percentiles (weight 46 kg, 78° pc, height 152 cm, 86° pc) and a relative microcephaly (51.5 cm, 18° pc). At the last visit, at the age of 14 years, the auxological parameters were within limits and more balanced (54 kg, 61° pc, 159 cm, 40° pc, HC 53 cm, 20° pc).

Peculiar facial traits included a low anterior hairline, sparse eyebrows, deep set eyes, short palpebral fissures, fine eyelashes, large nasal ridge, smooth philtrum, hypotonic mouth with small, conical and spaced teeth, and abnormal helix morphology. Furthermore, camptodactyly was noted on both hands. A few genetic analyses have been performed before: the target sequencing analyses of *FGFR1* and *FGFR3* genes and array-CGH, were negative. The clinical exome analysis performed in another Centre reported a non-paternal variant of unknown significance in the *KMT2C* gene (Chr7(GRCh38): g.152187342T>G, c.4928A>C, p.(Glu1643Ala)). *KMT2C* alterations have been previously related to Kleefstra Syndrome type 2 (MIM #617768).

## 3. Materials and Methods

In the context of the Drop by Drop research project, dedicated to clinical and genomic characterization of patients with Kleefstra diagnosis (FRRB Project 3441133), her genetic diagnosis was re-evaluated through whole-genome sequencing (WGS) duo analysis.

Once informed consent was obtained, DNA was extracted from leukocytes using standard procedure, and PCR-free whole-genome sequencing (Illumina Inc., San Diego, CA, USA) was performed on the proband and her father. Sequencing was performed using a 25B flow cell on an Illumina NovaSeq XPlus, with 150 bp paired-end reads (Illumina, PE 2x150). Alignment to reference genome GRCh38/USCShg38 and variant calling were performed with Illumina DRAGEN version 3.9.5. Variants were filtered according to autosomal recessive and dominant not-paternal models of inheritance and classified according to current guidelines [14,15] using the Illumina TruSight Software Suite version 2.6.3. Subsequently, TruPath™ Genome proximity mapped read technology (Illumina) for variants phasing.

Literature review was performed on Pubmed (https://pubmed.ncbi.nlm.nih.gov/, accessed on 3 February 2026), looking for English written papers reporting clinical data of patients harboring pathogenic alterations of the *PUS7* gene (search terms: “PUS7 AND syndrome”).

For the gestalt analysis, we used the Face2Gene software (F2G, FDNA Inc., Boston, MA, USA; https://www.face2gene.com, version 26.1.0, accessed on 24 April 2026), and Gestaltmatcher (https://db.gestaltmatcher.org/, algorithm version: v.0, accessed on 24 April 2026) [16], both freely available upon registration and consulted on 24 April 2026. In these deeper analyses, we included this patient’s photos along with those published of PUS7 patients. Only frontal images were selected, and any censored picture was excluded.

We hypothesized a convergence of IDDABS with ReNU syndrome and investigated it from clinical and dysmorphological perspectives. Particularly, despite the limitations imposed by the small sample size, we conducted preliminary gestaltic analyses through F2G research tool and Gestaltmatcher to compare the composite profile of PUS7 patients with that of ReNU syndrome, which was built using photographs from our internal database.

## 4. Results

### 4.1. Genetic Analyses

**Figure 1:**
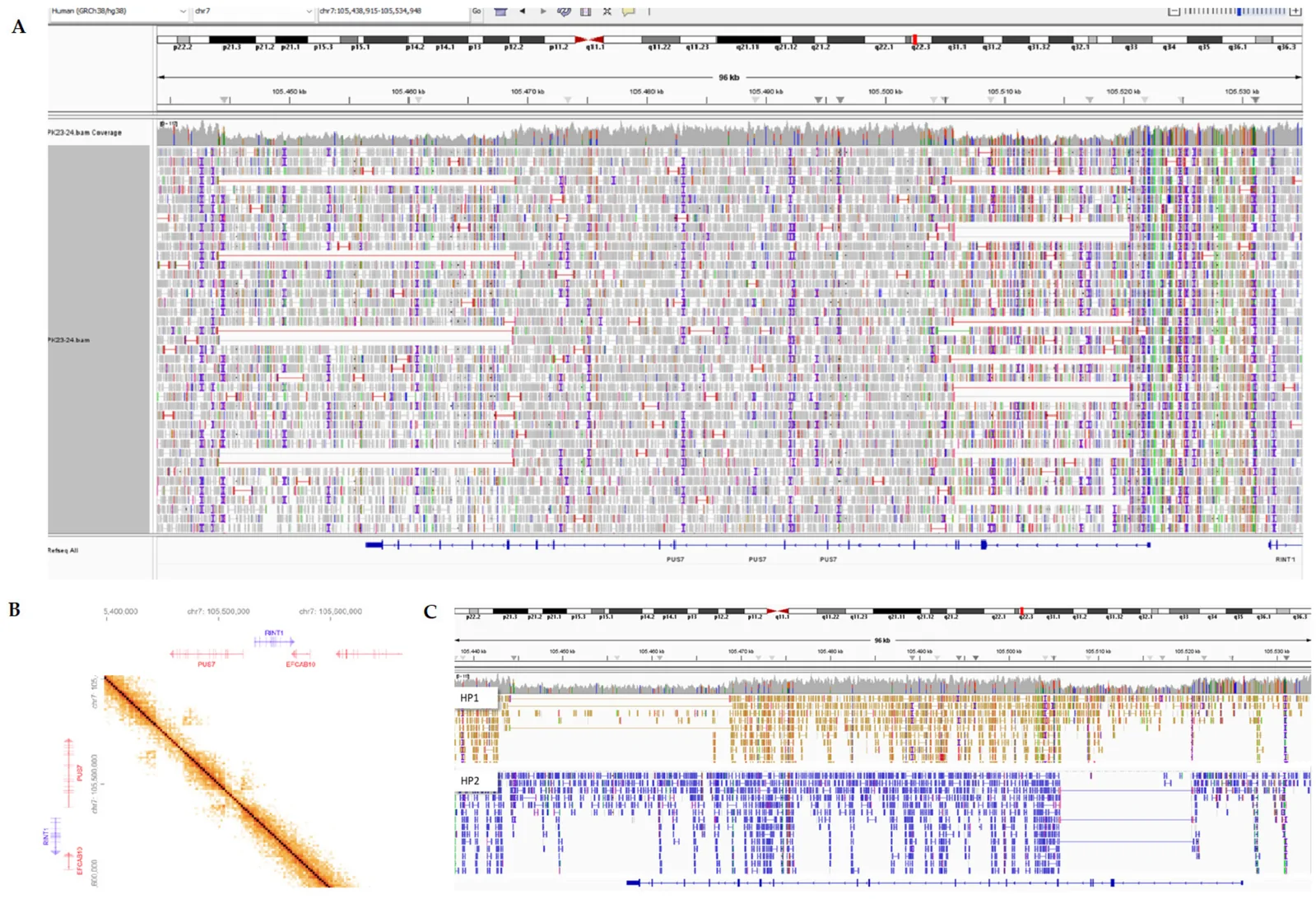
(**A**) Results of short-reads WGS duo analysis assessing in the proband the presence of two exonic deletions in the *PUS7* gene. (**B**) TruPath co-location plot, showing the two *PUS7* deletions. (**C**) Phasing of the two alleles by reads proximity mapping, showing the presence of *PUS7* deletions on the two different alleles (HP1 and HP2).

The WGS duo in depth investigation revealed two heterozygous deletions in the *PUS7* gene (MIM #616261). The first, seq[GRCh38]7q22.3(105444108_105468680)x1, Chr7(GRCh38): g.105444108_105468680, NM_019042.5:c.1399-217_*13682del, was paternally inherited. This heterozygous deletion spanned nearly 24.5 kb, involving the exon 12–exon 16 region of the *PUS7* gene. The second, seq[GRCh38]7q22.3(105505814_105520560)x1, Chr7(GRCh38):g.105505814_105520560, NM_019042.5: c.-33+1492_585+141del, was not paternally inherited. It was 14,7 kb in length, deleting the exon 2–4 region of the *PUS7* gene (see Figure 1A). No functionally similar deletions were found in the online databases (https://www.deciphergenomics.org/, https://dgv.tcag.ca/, https://clinicalgenome.org/, https://gnomad.broadinstitute.org/ SVs v4.1.0, accessed on 24 April 2026). According to the ACMG and ACGS guidelines [14,15], both variants scored 8 and were subsequently classified as likely pathogenic (assigned criteria: PVS1_strong; PM2_moderate, PM3_moderate). No other pathogenic variants were identified.

To establish the phase of the variants and exclude a de novo event on the paternal allele, TruPath™ Genome proximity mapped read sequencing (Illumina) has been performed and the presence of the two deletions in trans has been confirmed (see Figure 1B,C).

### 4.2. Literature Review

Data were collected from four papers collectively describing 13 individuals with pathogenic alterations in the *PUS7* gene [9,10,11,12,13]. One additional paper by Darvish and colleagues reported two siblings with a possibly overlapping but extremely aspecific neurodevelopmental phenotype, without microcephaly and short stature. The two reported brothers, born from consanguineous parents, harbored a homozygous missense variant in the *PUS7* gene (c.382G>A, p.Gly128Arg). However, the authors did not provide sufficient evidence of variant pathogenicity, and the report was subsequently excluded from our clinical review [17].

Similarly, four related patients reported by Naseer et al. in 2020 were excluded from the present review due to the dual diagnosis of IDDABS and Hyperlysinemia [18].

**Figure 2:**
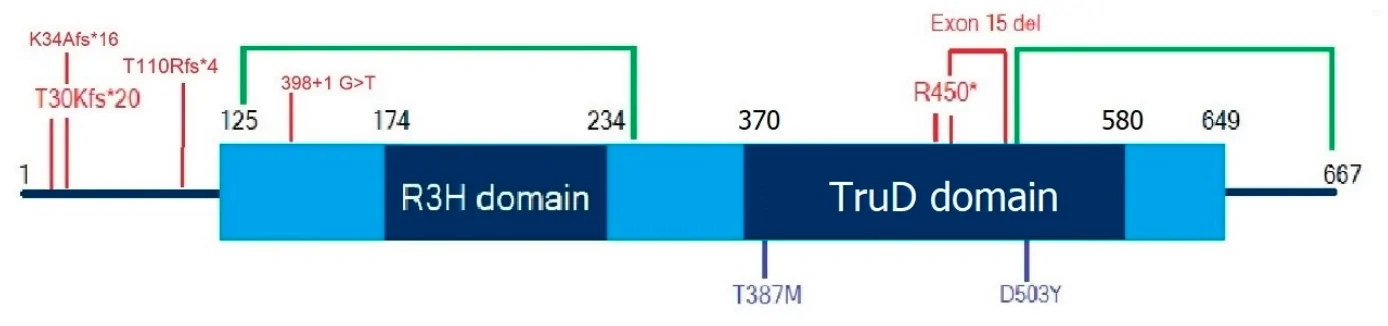
Graphical representation of causative alterations of IDDABS throughout the *PUS7* gene structure. In red are reported predicted haploinsufficient variants, in blue missense alterations. Green lines describe the extension of the two deletions identified in the present case.

Beyond the limitations given by the small number of diagnosed patients, causative alterations involving the *PUS7* gene do not appear to cluster within a specific gene region, nor recurrent variants have been described to date (see Figure 2 and Table 1). Conversely, several exonic deletions, occurring throughout the gene, have been reported in affected patients, as well as in the present case report (see Table 1).

Despite its relatively low HI score (Gnomad V4.0 LOEF = 0.6, CI: 0.48–0.76), a haploinsufficiency mechanism has been proposed to underlie the clinical phenotype related to the *PUS7* gene [10,11]. To date, only two missense variants have been demonstrated to express in a partial loss of protein function [10,11].

In Table 2, we report the clinical features of all 13 considered cases. IDDABS appears to be strongly characterized by neurocognitive impairment, reported in all affected patients. Poor growth and microcephaly are frequently described, but they are not evident in the present patient and in some others. Hearing loss and hypotonia have been reported in nearly half of PUS7 patients (see Table 2).

Driven by the gestaltic resemblance of IDDABS and ReNU syndrome, we decided to include in the present clinical review a comparative column aimed at exploring the convergence of these two rare conditions (see Table 2) [19]. The percentages shown in Table 2 should be interpreted with caution, as they are derived from different sources and are based on a small number of patients. Despite these limitations, the two disorders share several not specific clinical features (see Table 2), with the main exception of seizures, which have never been reported in IDDABS to date.

### 4.3. Facial Traits Analyses

Below, we reported all facial traits described in IDDABS patients and compared them to those reported in ReNU syndrome by Barbour and colleagues [20] (see Table 3). In *PUS7* patients, more recurrent characteristics include a prominent glabella, downslanted palpebral fissures, a smooth philtrum, and full lips with an everted lower vermilion; the mouth appearance is apparently quite similar to that depicted in ReNU.

**Figure 3:**
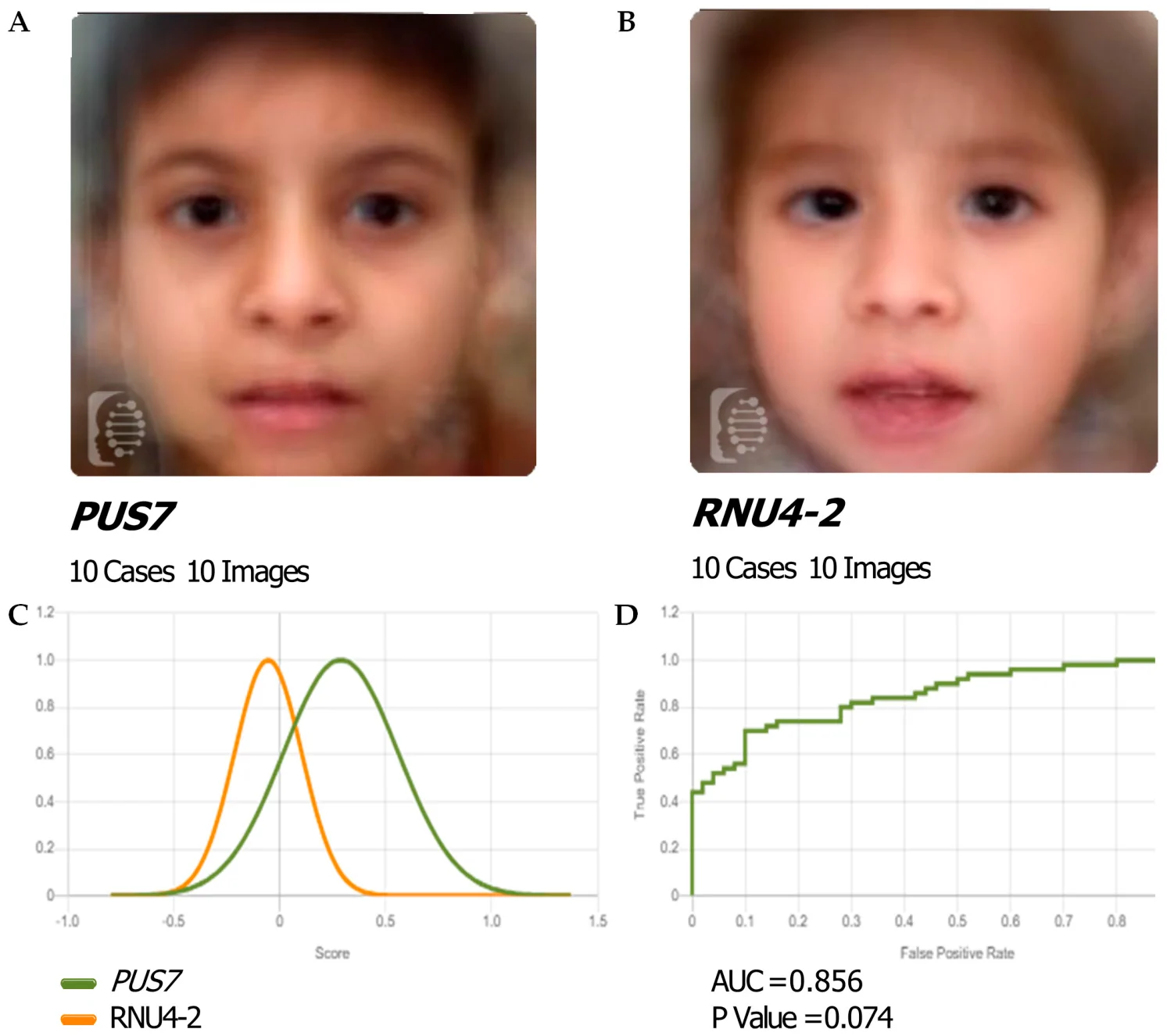
Composite profiles of *PUS7-* (**A**) and of RNU4-2- (**B**) related conditions created through the F2G research tool. (**C**) Results of binary comparison performed through Face2Gene tool between *PUS7* (green line) and ReNU syndrome (yellow line). (**D**) The ROC curve of the analysis and its *p* value.

Using published photographs, we were able to define a preliminary gestaltic profile of the *PUS7*-related condition. Through the F2G research tool, we investigated the phenotypic overlap between IDDABS and ReNU syndrome (see Figure 3) [20]. Although limited by small samples, the comparison of their dysmorphological profiles supports the hypothesis of phenotypical convergence. As reported in the F2G research analyses, the profiles of these two disorders can be well-distinguished by the algorithm (area under the curve, AUC = 0.856) but with a low significance (*p* value = 0.074) (see Figure 3C,D). The two composite profiles, despite the small sample size, appear quite similar in facts (see Figure 3A,B).

**Figure 4:**
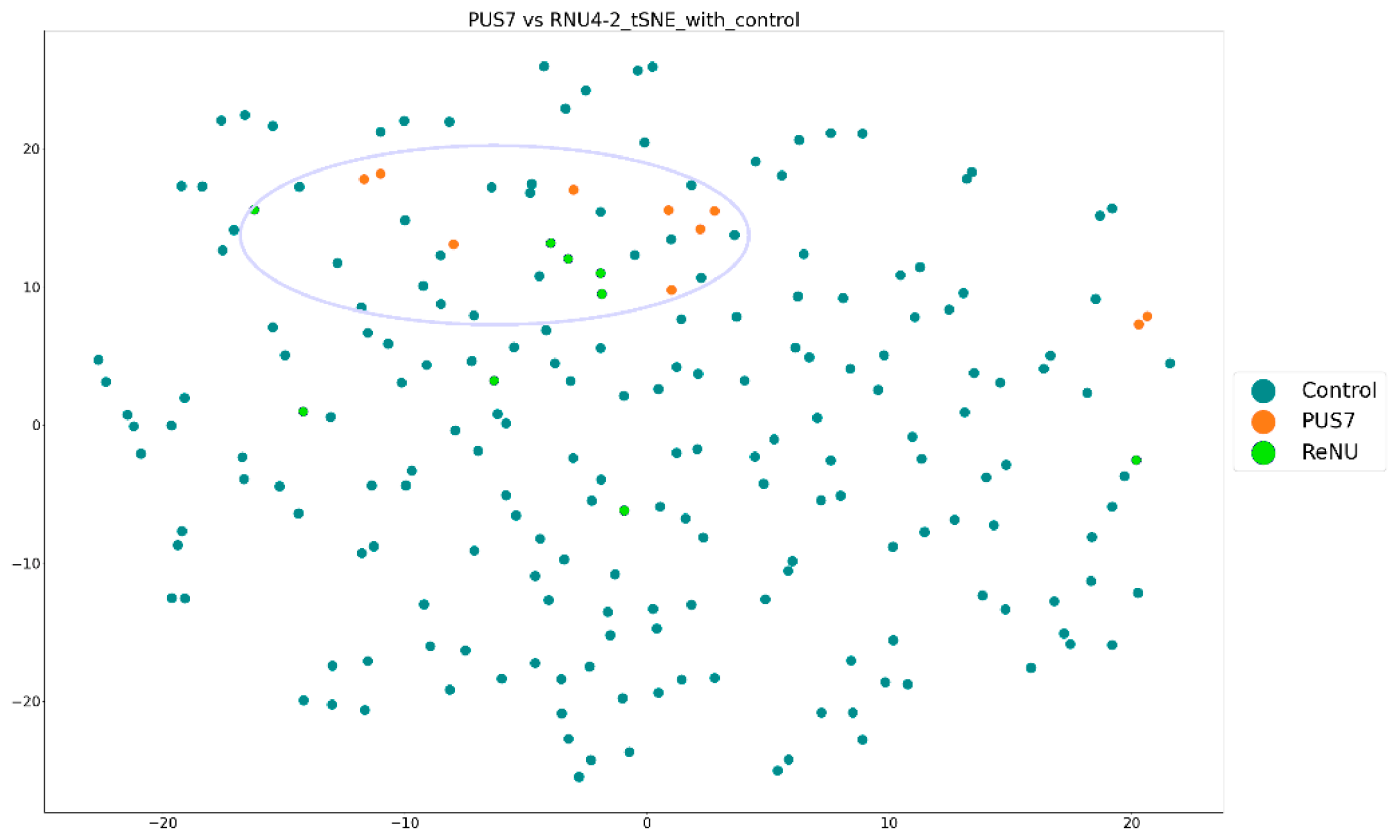
Results of comparison performed through Gestaltmatcher tool between *PUS7* (orange dots) and ReNU syndrome (blue dots). Each dot represents a single photograph. Into the light purple oval are contained most of the analyzed cases.

Finally, we measured the overlap between these two disorders using the Gestaltmatcher AI tool. This exploratory analysis, although constrained by the small size of both cohorts, further supports the existence of a phenotypical affinity between these two conditions (see Figure 4).

## 5. Discussion

Establishing the correct genetic diagnosis is a challenging journey that families undertake with the support of clinical geneticists and laboratory experts. This path is not exempt from pitfalls and false shortcuts, as in the case of the young girl described in the present report. A major barrier to achieving the final diagnosis is our incomplete understanding of genetics, specifically regarding variant interpretation and the phenotypic characterization of genetic disorders. As a matter of fact, every year, up to 300 novel diseases are discovered [21], but their rarity has a significant impact on our learning process regarding each of them. The present case report is a clear example of this scenario. In fact, the diagnosis of Kleefstra Syndrome type 2 was wrongly assumed after the identification of a missense variant of unknown significance in the *KMT2C* gene and this assumption was sustained by the scarcity of data concerning the related disease. Such a label might have been extremely difficult to withdraw due to the lack of data on the interpretation of the significance of *KMT2C* missense variants in NDD patients. Only in recent years has a more precise profile of the disorder related to *KMT2C* truncating variants been depicted [22]. In such a growing complexity, novel and ultrarare diseases represent a further challenge, both from the clinical and the molecular point of view.

Recent studies have reported different biallelic defects in RNA-independent pseudouridine synthases as involved in monogenic recessive neurodevelopmental disorders (*PUS1*, MIM: #608109, *PUS3*, MIM: #616283), providing insights into the role of pseudouridylation as a regulatory mechanism of gene expression and into its impact on protein synthesis [23,24,25]. In 2018, the biallelic loss of function of *PUS7* has been related to a novel disorder of RNA maturation, characterized by NDDs, short stature, and postnatal microcephaly [9,10,11,12,13]. As a result of genome-wide technologies, we were able to diagnose the reported patient with this extremely rare condition, caused by the presence of two different exonic deletions in compound heterozygosis. Given the family history, it was difficult to suspect a recessive disorder, and this posed a real challenge in determining the allelic phase of the deletions. The clinical characteristics of the proband fit well within the features of such an ultrarare disorder. She also underwent surgical correction for methopic craniosynostosis that may possibly explain the small head circumference reported in several *PUS7* patients. Within the context of such a rare disorder, the collected data suggest that individuals with IDDABS may present with hypoacusia and, in some cases, without growth defects, either in the neonatal and childhood setting, thus deviating from the disease’s acronym. Finally, we described for the first time the occurrence of heart anomalies and camptodactyly in a patient diagnosed with this ultrarare condition. Some peculiarities of the buccal region appear to be recurrent in the *PUS7* disorder, such as a smooth philtrum with full lips and an everted lower vermilion. Other commonly observed facial features include conical shaped teeth and simplified ear pinna (see Table 3).

During the preparation of this paper, Bergès and colleagues published a small cohort of 13 novel patients affected by IDDABS, recruited through an international collaboration via Genematcher [26]. No deletions similar to those identified in the present case are described in this paper. This cohort included five patients harboring monoallelic or biallelic missense variants, although no functional proof of pathogenicity was available. Despite the limitations associated with the small number of cases, this additional study suggests a higher prevalence of white matter abnormalities and hearing disorders among patients with *PUS7* defects. Clinodactyly and radioulnar synostosis were described in two other patients of this cohort [26]. From a dysmorphological point of view, the authors stated they did not recognize any clear gestaltic pattern, apart from a notable frequency of a smooth philtrum, aligning with our observations.

In the present study, we sought to more clearly delineate the facial features of the *PUS7*-related disorder, although our efforts were limited not only by the scarcity of available descriptions and photographs, but also by the ethnic diversity of the few reported patients. Using AI technologies and previously published photographs, we were able to reach a descriptive definition of the IDDABS gestaltic profile (see Table 3) and to create a tentative composite photograph of it (see Figure 3A). Furthermore, driven by our patient’s facial traits and by novel biological insights, we deepened the possible affinity between the *PUS7*-related disease and ReNU syndrome (MIM #620823). At present, this remains a hypothesis as the results cannot establish either the diagnostic similarity or biological relation between the PUS7-related disease and ReNU syndrome.

From a purely clinical perspective, ReNU and IDDABS share several but still not specific signs, including NDD, microcephaly, and poor growth, that often appear in the first stages of life (see Table 2) [19]. From a gestaltic perspective, our exploratory AI analysis of facial traits partially supports the hypothesized affinity between these novel but rare disorders (see Figure 3 and Figure 4). Given the small sample size, this hypothesis-generating analysis suggests that the two profiles are quite similar (see Figure 3). Nevertheless, the limited number of cases analyzed does not allow for any definitive conclusions regarding the phenotypic overlap between these disorders. What is even more intriguing is the concern that such a clinical convergence may rely on a biological interplay: in fact, both RNU4-2 and *PUS7* play roles in the intricate mechanisms that regulate RNA splicing [19,27]. While the function of RNU4-2 in this pathway is clearly evident, the definition of the precise role of pseudouridylation in several biological processes is still ongoing [28]. To further bridge the gap between these two ultrarare disorders, the role of *PUS7* in regulating gene expression via pre-mRNA alternative splicing has been recently described [29] and it has been already demonstrated that all the major spliceosomal snRNAs are pseudouridylated [30]. To add complexity to this already multifaceted scenario, a new recessive form of NDD has been recently discovered, associated with biallelic variants outside the 18-nucleotide ReNU syndrome region that cluster within other functionally important elements of U4 [31]. Our hypothesis regarding the possible clinical and biological overlap between *PUS7* syndrome and Re-NU syndrome must be confirmed by studies involving larger cohorts. Furthermore, future studies conducted on animal models could help clarify the role of the PUS7 protein in RNA modifications and splicing, thereby defining the nosological classification of this ultrarare disease more precisely [32,33]. Notably, RNA-modifying proteins are attracting increasing interest due to their recent implication in human diseases, whose pathogenic mechanisms are still far from being fully elucidated. What is clear to date is that aberrant RNA modification patterns affect gene expression and may be a major underexplored cause of human conditions.

The main limitation of this study is the small sample size of the PUS7 cohort, which is unavoidable given the extreme rarity of the syndrome and its recent discovery. This affects the reported prevalence of clinical features and the gestalt descriptions, and it also impacts comparisons between PUS7 syndrome and ReNU syndrome. For this reason, the results presented in this study should be interpreted with this caveat in mind. Similarly, the small number of available photographs, combined with their heterogeneity in terms of potential confounding factors such as age, sex, and ethnicity, limits the artificial-intelligence-based analysis.

## 6. Conclusions

This case report underscores the critical need to carefully evaluate the clinical implications of a presumed genetic diagnosis in patient management. In fact, when facing rare diseases, untagging is a more than challenging task, especially when the label arrives after an uncertain result of genetic tests. The main hurdles to reaching the correct genetic diagnosis are the unavoidable limits of our current knowledge in the field of genetics—which is still constantly evolving—as well as the huge clinical variability and the rareness of these conditions. In the presented girl, we reached an ultrarare alternative diagnosis as a result of genome-wide technologies. This novel description of a *PUS7*-related condition and our hypothesis-generating analysis of the literature and gestaltic profiles opens new horizons for the clinical phenotypes possibly associated with SPPs.

## Figures and Tables

**Table 1:** Genetic defects described in *PUS7*-diagnosed patients. Abbreviations: Co. Het: compound heterozygosis; Homo: homozygosis, N: number of cases, NA: not available.

Patient	Zigosity	GRCh38 (Chr7)	NM_019042.5	NP_061915.2
Present case	Co. Het	g.105505814_105520560del; g.105444108_105468680del	c.-33+1492_585+141del; c.1399-217_*13682del	p.?; p.?
[9] Family 1, N = 3	Homo	g.105508424_105508425del	c.89_90del	p.Thr30Lysfs*20
[9] Family 2, N = 2	Homo	g.105470738G>A	c.1348C>T	p.Arg450*
[9] Family 3, N = 1	Homo	NA	Exon 15 deletion	p.?
[10] Family 1, N = 2	Homo	g.105508182_105508185del	c.329_332delCTGA	p.Thr110Argfs*4
[10] Family 2, N = 1	Homo	g.105468355C>A	c.1507G>T	p.Asp503Tyr
[11] N = 2	Co. Het	g.105508114C>A; g.105481067G>A	c.398+1 G>T; c.1160 C>T	p.?; p.Thr387Met
[12] N = 1	Homo	g.105508182_105508185del	c.329_332delCTGA	p.Thr110Argfs*4
[13] N = 1	Homo	g.105508414_105508415del	c.99_100del	p.Lys34Alafs*16

**Table 2:** Clinical manifestations reported in patients with pathogenic *PUS7* alterations and in ReNU syndrome. Abbreviations: Abn.: abnormalities, ASD: autism spectrum disorder, DD: developmental delay, dis.: disorder, F: female, GERD: gastroesophageal reflux disease, GU: genitourinary, HC: head circumference, ID: intellectual disability, IUGR: intrauterine growth retardation; M: male; MRI: magnetic resonance imaging; NA: not available; perc.: percentage; RU: radio-ulnar; SD: standard deviations, SGA: small for gestational age, y: year.

Features	Present Case	[9] N = 6	[11] N = 2	[10] N = 3	[12] N = 1	[13] N = 1	Total N = 14 (Perc.)	ReNU [19]
Male/female	F	1 F; 5 M	2F	1F, 2M	M	F		
Age at last eval. (y)	14	2–18	2–4	8-16-14	3	3		
Pregnancy	AGA	3 SGA/6	2 SGA	1 SGA/3	SGA	SGA	7/14 (50)	IUGR 30%
Hypoglycemia	−	−	2/2	−	−	−	2/13 (17)	NA
Hyperuricemia	−	−	2/2	−	−	−	2/13 (17)	NA
Failure to thrive	−	−	2/2	−	−	−	2/14 (14)	52%
GDD/ID	+	6/6	2/2	3/3	+	+	14/14 (100)	100%
Hypotonia	+	1/6	2/2	-	+	+	6/14 (43)	64%
Brain MRI abn.	+	1/6	-	2/3	-	-	3/14 (21)	82%
Behaviour abn.	−	5/5	2/2	3/3	+	+	13/14 (93)	67%
Seizure	−	−	−	−	−	−	0/14 (0)	58%
Microcephaly	−	3/6	2/2	3/3	+	+	13/14 (93)	70%
Strabismus	+	0	½	0	−	−	2/14 (14)	56%
Hypoacusia	+	0	2/2	2/3	−	+	6/14 (43)	7%
Heart abn.	+	−	−	−	−	−	1/14 (7)	19%
Muscoloskeletal abn.	Pes planus	−	−	−	−	RU synostosis	2/14 (14)	NA
Poor growth	−	5/6	−	2/3	+	−	8/14 (57)	50%
Neurological abn.	−	−	1/2	1/3	−	−	2/14 (14)	NA

**Table 3:** Gestaltic traits described in IDDABS patients and in ReNU syndrome. NR: not reported. Numbers in brackets indicate the number of patients with this specific feature.

Facial Traits	Present Case	PUS7 Previous Cases [9,10,11,12,13] (N = 12)	ReNU Synd. [20]
Glabella	Prominent	Prominent (2)	NR
Eyebrows	Sparse	Arched (1) sparse (1)	Sparse
Eyes	Short palpebral fissures	Downslanted palpebral fissures (3), ptosis (2)	Deep set eyes
Nose	Broad tip	Upturned tip (5); broad nose (1)	Low-hanging columella
Philtrum	Smooth	Smooth (7)	Horizontally oriented
Malar hypoplasia	-	1	NR
Mouth	Full lips; everted lower lip	Full lips (4); everted lower lip (5); downturned (2)	Full lips
Teeth	Hypodontia, conical shaped teeth	Hypodontia (2); conical shaped teeth (1)	NR
Palate	-	Highly arched (1)	Highly arched
Chin	-	Micrognathia (3)	Retrognathia
Ears	Simple helix; low set	Prominent with simple helix (2); low set (2)	Large ears
Hands	Camptodactyly	Clinodactyly (2)	Clinodactyly and tapered fingers

## Data Availability

The datasets used and analyzed in the current study are available from the corresponding author upon reasonable request.

## Abbreviations

The following abbreviations are used in this manuscript:

Abn.	Abnormalities
AI	Artificial intelligence
ASD	Autism spectrum disorder
BL	Birth length
BW	Birth weight
CI	Confidence interval
DD	Developmental delay
Dis.	Disorder
F	Female
F2G	Face2Gene
GERD	Gastroesophageal reflux disease
GU	Genitourinary
HC	Head circumference
HI	Haploinsufficiency
ID	Intellectual disability
IUGR	Intrauterine growth retardation
IDDABS	Intellectual developmental disorder with abnormal behaviour, microcephaly, and short stature
LOEF	Loss-of-function observed/expected upper bound fraction
M	Males
MRI	Magnetic resonance imaging
NA	Not available
NDDs	Neurodevelopmental disorders
OFC	Occipitofrontal circumference
RU	Radio-ulnar
SD	Standard Deviations
SGA	Small for gestational age
SPPs	Spliceosomopathies
WGS	Whole genome sequencing
y	Year
